# Combating cough

**DOI:** 10.1038/npjpcrm.2016.12

**Published:** 2016-03-03

**Authors:** Alyn H Morice

**Affiliations:** 1 Respiratory Medicine, Castle Hill Hospital, Centre for Cardiovascular and Metabolic Research, Hull York Medical School, University of Hull, Yorkshire, UK

Thursday afternoon cough clinic is the highlight of my working week. There is always something unique and previously unrecognised, by me at least, even after running one for 30 years. In that time, cough has been transformed from a mere symptom of other diseases into a condition in its own right, a view recently endorsed by the European Respiratory Society who have accepted the definition of cough hypersensitivity as a ‘clinical syndrome characterised by troublesome coughing, often triggered by low levels of thermal, mechanical or chemical exposure’.^1^ Virtually all chronic cough occurs because of this hypersensitivity of the afferent nerves of the vagus. Patients are frequently amazed that you can predict that they have bouts precipitated by phenomena such as change in atmospheric temperature, strong smells and perfumes (it is always cheap perfume), and exposure to smoke and dust. The typical patient is a middle-aged woman. Women seem to have a heightened cough sensitivity, perhaps to protect them against aspiration during pregnancy.^2^ Associated conditions are irritable bowel syndrome and obesity.^3^ The relief the patient feels that someone understands the ‘sensitive throat’ from which they are suffering is palpable. The distress caused by chronic persistent coughing causes a decrement in quality of life greater than that seen in severe COPD or cancer.^4^

Over the years, our understanding of the diagnosis and treatment of chronic cough has also undergone radical change. Previously, chronic cough was thought to be because of three causes: asthma, GORD and postnasal drip. However, very few patients fit neatly into these boxes, and those who do show many atypical features from the classic diseases. Thus, patients who clearly had an asthmatic-type cough, which responded to inhaled corticosteroids, frequently had no wheeze or airflow obstruction, and thus the term cough variant asthma was invented.^5^ In others, there was not even bronchial hyper-responsiveness, and thus the definition was stretched further to include eosinophilic bronchitis.^6^ For those who did not fit into any of the boxes, we had to develop a term—idiopathic cough. The cough hypersensitivity syndrome does away with all of this confusion and gives the patient a reassuring and common diagnosis. However, what is the cause of the hypersensitivity?

Viruses hijack our cough reflex, giving rise to hypersensitivity in order to disseminate themselves to the next victim.^7^ Environmental insults, such as exposure to cold air, can cause inflammation leading to hypersensitivity. However, for the 1 in 10 of the world population who suffer from a chronic cough,^8^ this cannot be the answer. My view, and it is little more than that as I am unable to produce physiological proof, is that most chronic cough is a result of reflux, but not reflux as it is commonly understood.^9^ GORD is acid liquid reflux, but the reflux that causes the cough hypersensitivity is a non-acid gaseous mist, which we all produce, and in those who develop a cough it causes hypersensitivity of the airway nerves. I have therefore named this airway reflux.^10^ The reason why I am convinced this is true is that the patients tell us so. We have developed a validated questionnaire^11^ of 14 questions, which picks out the associated features, such as coughing with food or after meals, loss of voice, cough on lying down or on first rising in the morning. Each individual patient has a different profile of answers to these questions, but normal people score 4 out of 70. The upper limit of normal is 14, with most of the patients I see in the cough clinic having a score in the 30–60 s. The questionnaire is available on the website issc.info, and every patient who comes to see me fills it in beforehand. That way you do not waste time asking the questions to which the answer is negative. This is a fundamentally different problem from acid reflux, and it does not respond to anti-acid treatment. Promotility agents, such as metoclopramide, domperidone, baclofen and azithromycin, produce a successful response in the majority of patients.

In the linked paper, two physicians Richard Turner and Graham Bothamley^12^ report their experience from a cough clinic and find, unsurprisingly, that the overwhelming majority of patients could have been successfully treated in primary care had the referring physician taken the appropriate steps in management. This certainly concurs with my experience. A referral, such as ‘Please see this obese 50-year-old lady with a known hiatus hernia and previous irritable bowel syndrome’ is simply a waste of money. Frequently, Turner and Bothamley find that even the most basic of investigations have not been performed. All the guidelines say chest X-ray is mandatory, and although the yield in terms of diagnosing malignancy is low, the bronchial wall thickening of recurrent aspiration is not an unusual finding. It is these patients who are at greatest risk of having a decline in lung function. The majority of patients with chronic cough protect the airways with the cough reflex hypersensitivity. Others, however, particularly the older patients, aspirate leading to bronchial inflammation, which if they have been a smoker will be labelled as COPD, or even a frank bronchiectasis. It is these patients who are at most risk of progressive lung disease, and early vigorous treatment, up to and including fundoplication, is indicated.^13^ Turner and Bothamley have applied the existing guidelines to their patients and found that had these procedures been applied in primary care before referral the majority of patients would have been successfully managed. The guidelines are, however, well out of date, and despite the best efforts of specialists to update them to include modern evidence, just as with the NICE COPD guidelines, we are left to make up our own minds in the modern world.

In exact parallel to the revolution that is occurring in COPD, there is a realisation that in chronic cough treatment is governed by the type of inflammation that is occurring in the airways. Our previous simple paradigm that asthma was an extrinsic allergic condition, although true, is only the tip of the iceberg when we are dealing with eosinophilic, TH2-type inflammation with the lungs. More recent studies have shown that non-allergic, innate, mechanisms caused by epithelial damage can precipitate, in predisposed individuals, to an eosinophilic-type reaction.^14^ Although it is possible to use techniques such as induced sputum and exhaled nitric oxide to determine which of these patients sitting in front of you have this eosinophilic-type inflammation, simply looking at the blood eosinophilic count from the historical records may be just as valuable. This is certainly what I do in the clinic. If the eosinophil count is above 0.3, either repeatedly or on an occasional basis, then I would classify the patient as having an asthmatic cough, and anti-asthma treatment is indicated. Because the inflammation is more deep-seated, inhaled steroids may be only partially effective.^15^ To establish the diagnosis, a prednisolone trial may be indicated, and because the particular lymphocyte involved in this form of innate immunity is packed with leukotriene receptors, the anti-leukotriene drugs, such as montelukast, can be highly effective.^16^

Therefore, I agree with doctors Turner and Bothamley that the overwhelming number of patients with chronic cough should be successfully managed in primary care. A chest X-ray and spirometry with a Hull Airways Reflux Questionnaire will establish the diagnosis in the majority of patients. The type of inflammation, eosinophilic or not, can be determined in the office from the historical blood counts. Treatment can then be either directed against the eosinophilic inflammation, the oesophageal dysmotility or both. If we do this, my clinic will become even more interesting, as the weeping patients who declare ‘doctor you are the first one who has understood’ will have already been successfully managed and I will be left with the exotic yellow nail syndrome, mononeuritis multiplex or, as last Thursday, the lady who watched TV with a parrot on her shoulder.
